# Temporomandibular Disorders and Bruxism Outbreak as a Possible Factor of Orofacial Pain Worsening during the COVID-19 Pandemic—Concomitant Research in Two Countries

**DOI:** 10.3390/jcm9103250

**Published:** 2020-10-12

**Authors:** Alona Emodi-Perlman, Ilana Eli, Joanna Smardz, Nir Uziel, Gniewko Wieckiewicz, Efrat Gilon, Natalia Grychowska, Mieszko Wieckiewicz

**Affiliations:** 1 Section of Dental Education, Department of Oral Rehabilitation, The Maurice and Gabriela Goldshleger School of Dental Medicine, Tel Aviv University, Tel Aviv 6139001, Israel; dr.emodi@gmail.com (A.E.-P.); elilana@tauex.tau.ac.il (I.E.); niruziel@gmail.com (N.U.); gilon.efrat@gmail.com (E.G.); 2 Department of Experimental Dentistry, Wroclaw Medical University, 50-425 Wroclaw, Poland; joannasmardz1@gmail.com; 3 Department and Clinic of Psychiatry, Medical University of Silesia, 42-612 Tarnowskie Gory, Poland; gniewkowieckiewicz@gmail.com; 4 Department of Prosthetic Dentistry, Wroclaw Medical University, 50-425 Wroclaw, Poland; natgrychowska@gmail.com

**Keywords:** COVID-19, SARS-CoV-2, coronavirus pandemic, temporomandibular disorders, bruxism, orofacial pain

## Abstract

Background: In late December 2019, a new pandemic caused by the SARS-CoV-2 (Severe Acute Respiratory Syndrome Coronavirus 2) infection began to spread around the world. The new situation gave rise to severe health threats, economic uncertainty, and social isolation, causing potential deleterious effects on people’s physical and mental health. These effects are capable of influencing oral and maxillofacial conditions, such as temporomandibular disorders (TMD) and bruxism, which could further aggravate the orofacial pain. Two concomitant studies aimed to evaluate the effect of the current pandemic on the possible prevalence and worsening of TMD and bruxism symptoms among subjects selected from two culturally different countries: Israel and Poland. Materials and Methods: Studies were conducted as cross-sectional online surveys using similar anonymous questionnaires during the lockdown practiced in both countries. The authors obtained 700 complete responses from Israel and 1092 from Poland. In the first step, data concerning TMDs and bruxism were compared between the two countries. In the second step, univariate analyses (Chi^2^) were performed to investigate the effects of anxiety, depression, and personal concerns of the Coronavirus pandemic, on the symptoms of TMD, and bruxism symptoms and their possible aggravation. Finally, multivariate analyses (logistic regression models) were carried out to identify the study variables that had a predictive value on TMD, bruxism, and symptom aggravation in the two countries. Results: The results showed that the Coronavirus pandemic has caused significant adverse effects on the psychoemotional status of both Israeli and Polish populations, resulting in the intensification of their bruxism and TMD symptoms. Conclusions: The aggravation of the psychoemotional status caused by the Coronavirus pandemic can result in bruxism and TMD symptoms intensification and thus lead to increased orofacial pain.

## 1. Introduction

Temporomandibular disorders (TMD) are a group of conditions that cause pain and dysfunction of the masticatory muscles, the temporomandibular joints (TMJs), and associated structures. The most common features of TMD are regional pain, limited jaw movements, and acoustic sounds from TMJs during motions [1]. The prevalence of TMD in the general population is estimated at about 10–15% [2,3,4], and these conditions affect women more frequently than men. Psychosocial factors, such as anxiety, stress, depression, coping strategies, and catastrophizing, may influence the onset of pain, as well as precipitate or prolong the TMD pain [5,6,7,8]. The International Association for the Study of Pain (IASP) reported that TMD-related facial pain occurs in 9–13% of the general population, while only 4–7% seek treatment. The TMD-related pain may also affect the daily activities, physical and psychosocial functioning, and quality of life of the affected individuals [9].

Bruxism is a repetitive jaw muscle activity characterized by clenching or grinding of the teeth, and/or bracing or thrusting of the mandible [10]. It can act as a potential risk factor for several negative consequences of health such as masticatory muscle pain, oral mucosa damage, mechanical tooth wear, and failures of prosthodontic constructions [11,12,13]. This condition is divided into sleep bruxism (SB) awake bruxism (AB). The prevalence of SB is estimated at about 16% among young adults and at 3–8% among adults, while the prevalence of AB in the general population is estimated at about 22–30%. Both forms of bruxism men and women equally [14].

Psychosocial factors, such as stress and anxiety, have been indicated as associated with both SB and AB [15,16,17,18,19,20]. However, the latest research showed that self-reported perceived stress was not correlated with the intensity of SB [21].

In late December 2019, a new unfamiliar and threatening pandemic called COVID-19 (Coronavirus 2019 disease), which is caused by the SARS-CoV-2 (Severe Acute Respiratory Syndrome Coronavirus 2) infection, began to spread around the world. Due to almost complete uncertainty about the ways of virus spread [22] and the appropriate modes of treatment, insufficient availability of health services, and no existing vaccine or efficient drug for treatment, most countries adopted the policies of social distancing and partial to total lockdown.

The situation continued, and within weeks, routine life was drastically altered. This gave rise to severe health threats, economic uncertainty, and social isolation, causing potential deleterious effects on the physical and mental health of the people. The common psychological responses of individuals to the Coronavirus pandemic included stress, anxiety, and depression [22]. All these are capable of influencing the oral and maxillofacial syndromes, such as TMD and bruxism, which could further aggravate the orofacial pain [23].

Studies aimed to: (i) evaluate the effect of the current Coronavirus pandemic on the possible prevalence and worsening of TMD and bruxism symptoms, among subjects selected from two culturally different countries: Israel and Poland; and (ii) to define the predictors of TMD and bruxism during the lock down periods, in the above countries.

## 2. Materials and Methods

Studies were conducted as cross-sectional online surveys using anonymous questionnaires. The final questionnaire was compiled from tools commonly used with regard to TMD, bruxism, anxiety and depression (3Q/TMD, possible/probable bruxism, and Patient Health Questionnaie-4, as detailed below), and specific questions referring to demographics, concerns specific to the Coronavirus, media consumption, etc. The latter were agreed upon, and tested for content validity, by a group of subject matter experts (SMEs). The group consisted of four dentists (AE-P, IE, NU, and EG) who work at the Tel Aviv University School of Dental Medicine and have vast clinical and academic experience in working with patients suffering from TMD and bruxism. Each SME proposed questions for the study and, following discussions, the final questions were agreed upon. The questionnaire was compiled in Hebrew and translated to Polish by the Polish group. The surveys were carried out one month after the start of the total lockdown periods in each of the countries.

### 2.1. Population

The questionnaire was distributed through the internet (in Hebrew in Israel, in Polish in Poland).

In Israel, the study questionnaire was posted on SurveyGizmo (https:www.mysurveygizmo.com/s3) and distributed through mailing lists of dental clinics and social media (e.g., Facebook and WhatsApp).

In Poland, the questionnaire was posted on Reddit, an American social news aggregation platform that allows the users to interact on community-created discussion forums, and on r/Polska sub-reddit.

In both countries, the responses were given anonymously by the participants.

Studies were conducted in full accordance with the World Medical Association Declaration of Helsinki. In Israel, all the study procedures were approved by the Ethics Committee of the Tel Aviv University in Israel (ID: 0001332-1). In Poland the Bioethical Committee of the Wroclaw Medical University approved the study protocol (ID: KB-302/2020). Informed consent was obtained from all the subjects as required.

### 2.2. Instruments

The following data were collected from the participants:

1. Demographic and general information: This included the consent to participate in the study, age, gender, and conjugal status (with partner and children, with partner but no children, with children but no partner, with roommate, alone).
2. Concerns specific to the Coronavirus: These included worries about the risk of being contaminated (yes/no), and about the financial aspects, physical health, mental health, and relationship with relatives and friends (ranging from 1—not at all to 5—very worried).
3. TMD screening: The 3Q/TMD questionnaire, which is a reliable and acceptable tool for screening the TMD conditions, was used for collecting data [24,25]. The questionnaire has an excellent negative predictive value and is regarded as a valid tool for screening [24,25]. It asks about the existence of pain in the temple, face, and jaw during mouth opening or chewing, and whether there is an experience of jaw locking. A positive response to one of these confirms the presence of TMDs.
4. Possible/probable AB: An accepted way to assess possible AB and/or SB is the use of a self-report questionnaire [12,17,26]. The questions are related to awareness (by self or being told by others) of grinding, clenching, and holding the teeth together and/or tightening the masticatory muscles during the day (scale ranging from 0—never to 4—all the time). A positive answer to one of these (either than “never”) confirms the presence of “possible AB”. An additional positive response to the question that refers to “being told by a dentist that you clench/grind your teeth” confirms the presence of “probable AB” [10,27].
5. Possible/probable SB: It is assessed through the question, “Do you know or have been told that you clench or grind your teeth while you sleep?” A scale ranging from 0 (never) to 4 (4–7 nights/week) is used for this assessment. Any score above 0 (never) confirms the presence of “possible SB”. An additional positive response to the question that refers to “being told by a dentist that you clench/grind your teeth” confirms the presence of “probable SB” [10,27].
6. Possible aggravation of symptoms associated with TMDs and bruxism (“since the beginning of the Coronavirus confinement do you feel any changes in…etc.”). The evaluated symptoms referred to: (i) pain in temple, face, jaw or jaw joint, pain at mouth opening or chewing and jaw locking (for TMD); (ii) headache during the day in the temple area, exacerbation in pain levels during the day and change in the temple pain upon functioning (for TMD and AB); and (iii) difficulties in mouth opening upon awaking, jaw and/or muscle stiffness upon awaking and temple headache that is reduced after some time (for SB) [28]. The scores were as follows: no change, slight aggravation, significant aggravation, and improvement.
7. Anxiety and depression: The Patient Health Questionnaire-4, a brief screening tool, is used for assessing anxiety and depression [29]. The total score of this questionnaire ranges from 0 to 12, and the conditions are usually evaluated using the following cut-off scores: 0–2, normal; 3–5, mild; 6–8, moderate; 9–12 severe [29]. The questionnaire also allows performing a separate evaluation for anxiety and depression.
8. Media consumption: Report of news consumption concerning the Coronavirus pandemic through television, internet, and/or social media was also assessed (scale ranging from 1—not at all to 4—all reports/all the time).

All questions were formulated in a first person voice (referring to self), and referred to the last 30 days, namely, to the period of the lock down.

The surveys were open to anyone who entered the SurveyGizmo (https:www.mysurveygizmo.com/s3) site and/or the Facebook and/or WhatsApp apps (in Israel) or the r/Polska sub-reddit in Poland.

In Israel, complete lock down was imposed on 19 March 2020. Data were collected from 16 April (namely, four weeks after the beginning of the complete lock down) to 20 May 2020. In Poland, complete lock down was imposed on 31 March 2020. Data were collected from 29 April (four weeks after the beginning of the lockdown) to 3 May 2020.

### 2.3. Statistical Analysis of Data

Data analysis was performed using STATISTICA PL Version 12 software (Tulsa, OK, USA), with the level of significance set at *p* < 0.05. In the first step, the data concerning TMDs, AB, and SB were compared between the two countries (descriptive analyses). In the second step, univariate analyses (Chi^2^) were performed to investigate the effects of anxiety, depression, and personal concerns of the Coronavirus pandemic (being contaminated, being influenced financially, experiencing negative effects on physical and/or mental health and on the relationship with relatives and friends) on the symptoms of TMDs, SB, and AB and their possible aggravation. Finally, multivariate analyses (logistic regression models—binomial logit models) were carried out to identify the study variables that had a predictive value on the symptoms of TMDs, AB, and SB and their aggravation.

## 3. Results

In Israel, a total of 867 subjects responded to the questionnaire, out of whom 80.74% (*N* = 700) fully completed it. In Poland, a total of 1096 subjects responded to the questionnaire, of which 99.63% (*N* = 1092) fully completed it.

The age groups of participants were defined according to “young adults” (age of 18–35 years) and “adults” (36–56 years old) as accepted in the literature [30]. Some significant differences existed between the two populations with regard to gender and age groups (Table 1 and Table 2).

The Polish population had more females (*p* < 0.05), and the participants were significantly younger compared to their Israeli counterparts (*p* < 0.05).

Due to these significant differences in age and gender between the studied populations, comparisons were carried out separately for males and females, categorized into predefined age groups.

### 3.1. Descriptive Analyses—TMDs, Possible/Probable AB, and Possible/Probable SB

1. TMD screening: The results showed that the odds of occurrence of TMDs among the Polish young adult and adult age groups (18–35 years and 36–55 years) were significantly higher for both males and females as compared to the Israeli groups (odds ratios ranged from 3.04 to 5.37). However, no such differences were observed for the elderly group (>56 years) between the populations (Table 3).

2. Possible/probable AB: Similar results were found for possible/probable AB. The odds of occurrence of these conditions among the Polish participants were significantly higher in general than among the Israeli participants (except the young and elder males), with the odds ratios ranging between 2.51 and 6.41 (Table 4).

3. Possible/probable SB: The findings for possible/probable SB were also consistent. The odds of occurrence of these conditions among the Polish subjects (except for males in the two higher age groups) were similar to those of the Israeli subjects, with the odds ratios ranging from 1.4 to 3.99 (Table 5).

### 3.2. Aggravation of AB, SB and TMD Symptoms

Almost half (48.8%) of the Poles reported experiencing at least once a week pain in temple, face, jaw or jaw joint during the past 30 days, namely, since the beginning of the lockdown. A total of 247 individuals (22.6%) declared pain during mouth opening or chewing and 101 (9.2%) jaw locking or getting stuck at least once a week. Among the Israelis, the numbers were 166 (23.7%), 91 (13.0%), and 35 (5.0%), respectively.

Among the Polish responders, 372 (34%) reported TMD symptoms aggravation, 372 (34%) AB aggravation, and 311 (28%) SB aggravation. Among the Israeli responders, 107 (15%) reported TMD symptoms aggravation, 111 (16%) AB symptom aggravation, and 94 (13%) SB symptom aggravation.

Both in Israel and in Poland, females reported more symptoms of TMD, AB, SB and symptom aggravation, than males (Chi^2^, *p* < 0.05 for all). However, further logistic regression analyses, performed among Israeli population (see below), rejected gender as a predictor of SB. Distributions of TMD, AB, SB among males and females in Poland and in Israel are presented in Table 3, Table 4 and Table 5.

### 3.3. The Effect of Conjugal Status

Significant relationships were observed between subjects’ conjugal status and TMD aggravation, AB aggravation and SB aggravation among the Polish responders (Chi^2^, *p* < 0.05, for all). Respondents living with a roommate or sharing apartment with a partner, reported more TMD and AB aggravation than those living with a spouse without children (Chi^2^, *p* < 0.001 for both). They also reported higher SB symptom aggravation than those with children but with no partner or spouse (*p* < 0.001).

In Israel, no differences in TMD, AB. and SB symptom aggravation were observed among subjects with different conjugal status.

### 3.4. The Effect of Demographic Data on Anxiety and Depression

In Poland, anxiety was more frequent among females than males (Chi^2^, *p* < 0.05). Additionally, a significant relationship was found between subjects’ conjugal status and depression (*p* < 0.05). Depression was more often among respondents living with a roommate or sharing an apartment with a partner than among responders living with spouse and children (*p* < 0.001). There were no significant relationships between gender and depression or age and depression, between age and anxiety and between conjugal status and anxiety.

In the Israel, anxiety and depression were more frequent among females than males (Chi^2^, *p* < 0.05). No relationships between conjugal status and depression or anxiety, and between age and depression were detected. Anxiety was more frequent among young adults (18–35 years) than among the elderly group (>56 years) (Chi^2^, *p* < 0.001).

### 3.5. Effect of Anxiety, Depression, and Personal Concerns on TMD, SB, and AB (Chi^2^)

1. TMD: The presence of anxiety, depression, or personal concerns significantly increased the odds of occurrence of TMDs among both populations. The odds ratio ranged between 1.32 (concerns of being contaminated by the virus) and 2.75 (anxiety) for the Polish subjects, while it ranged between 1.46 (concerns about personal finances due to the pandemic) and 6.4 (anxiety) for the Israeli population.

2. Possible/probable AB: The presence of anxiety, depression, and personal concerns significantly increased the odds of occurrence of possible/probable AB among both populations. The odds ratios ranged from 1.45 (concerns of being affected financially, for Polish subjects) to 2.85 (anxiety, for Israeli subjects).

3. Possible/probable SB: Mixed results were observed for possible/probable SB. In Poland the odds ratios ranged from 1.34 (concerns of being affected mentally) to 1.84 (anxiety). No effect was observed for the concerns regarding personal finances or depression. Among the Israeli subjects, the odds ratios ranged from 1.38 (worries of being affected financially) to 2.27 (anxiety). No effect was observed for worries of being contaminated by the virus.

### 3.6. Effect of Anxiety, Depression, and Personal Concerns on the Possible Aggravation of TMD, SB, and AB Symptoms (Chi^2^)

1. Aggravation of TMD symptoms: Anxiety, depression, and personal concerns significantly increased the odds of aggravation of TMD symptoms in both populations. The odds ratios ranged from 1.58 (concerns regarding personal finances, for Polish subjects) to 3.03 (anxiety, for Polish subjects).

2. Aggravation of possible/probable AB symptoms: The obtained results were similar with regard to the aggravation of AB symptoms. The odds ratios ranged from 1.36 (concerns regarding personal finances, for Polish subjects) to 3.95 (anxiety, for Israeli subjects).

3. Aggravation of possible/probable SB symptoms: Similar results were observed for the aggravation of SB symptoms. The odds ratios ranged from 1.60 (concerns regarding personal finances, for Polish subjects) to 3.32 (anxiety, for Israeli subjects).

### 3.7. Multivariate Analyses (Logistic Regression)

1. TMD: The best predictors of TMD in Poland were female gender, anxiety, and personal concerns (worries of being contaminated by the virus and about the pandemic’s effect on mental health) (Table 6). Aggravation of TMD was best predicted by female gender, worries of being contaminated, use of social media to look for information about the pandemic, and worries about the pandemic’s effect on mental health (Table 7).

On the other hand, the only significant predictor of TMDs in Israel was anxiety (Estimate: 0.917, S.E.: 0.107, Wald: 73.922, df: 1, odds ratio 6.25, 95% confidence interval 4.11–9.49).

The best predictors of TMD aggravation in Israel were female gender, concerns about the pandemic’s effect on the relationship with family and friends, and anxiety (Table 8).

2. Possible/probable AB: In Poland, the best predictors of possible/probable AB were female gender, concerns of being contaminated by the virus, and concerns about the pandemic’s effect on mental health (Table 9). The aggravation of AB was best predicted by concerns about being contaminated by the virus, anxiety, concerns of the pandemic’s effect on physical and/or mental health, and use of social media for obtaining information about the pandemic (Table 10).

In Israel, the best predictors of possible/probable AB were female gender, depression, concerns regarding personal finances, and anxiety (Table 11). The aggravation of AB was best predicted by female gender, concerns about the pandemic’s effect on the relationship with relatives and friends and on mental health, and anxiety (Table 12).

3. Possible/probable SB: In Poland, the best predictors of possible/probable SB were female gender, worries of being contaminated by the virus, and anxiety (Table 13). The aggravation of SB was best predicted by female gender, worries of being contaminated by the virus, anxiety, use of social media, and concerns of the pandemic’s effect on mental health (Table 14).

In Israel, possible/probable SB was best predicted by anxiety and concerns regarding the pandemic’s effect on the relationship with relatives and friends (Table 15). The aggravation of SB was best predicted by female gender, anxiety, and concerns about mental health (Table 16).

## 4. Discussion

The two studies, carried out in two different countries, used similar tools and collected data at similar points in time, as far as the pandemic progression and lock down periods are concerned. In Israel, data collection started four weeks after the beginning of a total lockdown in the country. Schools, kindergartens, and universities were closed. Leaving home for a distance more than 100 m was prohibited, except for emergency, buying basic products, or work in vital posts (specifically defined by the government). All nonemergency medical and dental treatments were stopped. Shops, restaurants, and most public places were shut down. Personal contact with family members not cohabitating in the same home and/or with friends was forbidden. Similarly, in Poland, data collection started four weeks after the beginning of a total lockdown in the country, when the country was practicing an almost complete lockdown with similar regulations as mentioned above for Israel (with minor exceptions, e.g., there were no limitations on the distance of leaving home). Although the studied populations in Poland and in Israel were not similar, age- and/or gender-wise, the similarity in research tools and in the point in time allows us to evaluate some interesting differences between the two societies.

The first emerging finding of the two studies is that significant differences existed in the odds of occurrence of bruxism (AB and SB) and TMD between the Polish and Israeli populations during the lock down periods in the two countries. Except in a few cases (higher age group), the odds in Poland were found to be higher by several hundred percent than those in Israel.

In the general population, the prevalence of bruxism is estimated at 8–31% and tends to decrease with age [31]. SB prevalence is about 16% among young adults and 3–8% among adults, while the AB prevalence in the general population is 22–30% [14]. Even the reported prevalence of bruxing activities has a large range (2.7–57.3% for AB, 4.1–59.2% for SB) [26]. When considering TMD, it is believed that about 75% of the general population may experience at least one TMD-associated sign during their lifetime and about 33% have at least one TMD symptom at each time [32]. The differences origin mostly in different modes of measuring.

Regretfully, accurate data on possible differences in pre-pandemic occurrence of bruxism in the Polish versus Israeli populations are not available. However, some studies from Poland and from Israel suggest that the occurrence of TMD in the Polish population may differ from that in the Israeli population. Wieckiewicz et al. reported that 54% of Polish university students present TMD symptoms [33]. In another study, the same group of authors reported that 56% of participants were diagnosed with pain-related TMD after a clinical examination [34]. In Israel, Winocur et al. reported that 37% of individuals had at least one TMD symptom [35]. Thus, the differences between countries, observed in the present study, may be due to several reasons. First, the higher findings of TMD in the Polish populations may have been there before the pandemic [33,34,35]. Possibly, the increase in anxiety/depression in both countries affected TMD and bruxism in both countries in a proportional manner. Additionally, the differences in the demographic properties of populations were significant, a fact that might have affected the results.

As both bruxism and TMD can be caused and intensified by psychologic factors [8,31], the differences in their prevalence during the pandemic could have resulted from the psychological differences between the participants. These, in turn, may result from ethnic, socioeconomic, political, and cultural differences between the Polish and Israeli societies [36,37]. These factors could have potentially modulated the psychoemotional status of the participants, influenced their coping strategies during the Coronavirus pandemic, and in turn increased the prevalence of both bruxism and TMD in Poland. However, this issue needs a further study focused on differentiating between the populations.

It should also be emphasized that TMDs are closely associated with orofacial pain. The IASP reported that TMD-related facial pain occurs in 9–13% of the general population. As TMD-related pain can affect the daily activities, physical and psychosocial functioning, and quality of life of the affected individuals, such a relationship could play an important role during the COVID-19 pandemic [9]. Increased psychosocial distress during the pandemic can exacerbate the TMD symptoms, including those associated with orofacial pain, which in turn may further negatively affect the patients’ psychoemotional status.

When the effects of anxiety, depression, and personal concerns on TMD, SB, and AB, and the aggravation of their symptoms (pain in temple, face or jaw, pain when opening mouth, sticking of jaw, headache, difficulty in mouth upon awaking, and stiffness in jaw upon awaking, etc.) were analyzed, some similarities were observed between the countries. Although the odds of occurrence of TMD, SB, and AB in Poland were by far higher than in Israel, the effects of emotional factors and of personal concerns on the associated symptoms and their aggravation were found to be similar in both countries. Anxiety, depression, and worries regarding finances, health and relationships significantly increased the odds of occurrence of bruxism and TMD in both the Polish and Israeli societies (with some minor exceptions).

Apparently, anxiety, depression, and personal worries evoked by the Coronavirus pandemic increased the prevalence of TMD and bruxism. This is in line with the literature results, that anxiety, stress, depression, coping strategies, and catastrophizing may precipitate or prolong the TMD pain [2,3,4,5,6,7,8], and that psychosocial factors are associated with both forms of bruxism [13,14,16,17,18,19,20]. When the pandemic situation kept changing rapidly from day to day, uncertainty and worries about the present and future were common and unavoidable [38,39]. Moreover, subjects had to stay home and many were unemployed, with the media constantly broadcasting apocalyptic news. Under such conditions, a significant increase in the odds of occurrence of TMD, SB, and AB is not surprising.

The one prominent difference was observed between the studied populations. The studies show that unlike the Polish participants, the worry of being contaminated by the virus did not increase the odds of occurrence of AB and SB, or aggravate the symptoms of the conditions (TMD, SB, and AB) among the Israeli subjects. This may be explained by the advanced and generally good public health services available in Israel. All the Israeli citizens have governmental health insurance and are entitled to all the necessary health services with no extra costs (besides a mandatory monthly fee). Furthermore, hospitals are considered to meet high medical standards, and medical personnel are required to be well trained. In Poland, citizens’ trust in national healthcare system is limited [40].

Logistic regression models used in this study for identifying the variables that can serve as significant predictors of TMD, SB, AB, and/or the aggravation of their symptoms, showed that female gender was significant in most of the calculations. In Poland, female gender played a significant role in predicting the presence of TMD, AB, and SB, as well as the symptom aggravation, while in Israel this factor played a significant role in predicting the presence of AB (but not TMD or SB) and the aggravation of TMD, SB, and AB symptoms.

The role of gender is expected because most of the TMD patients worldwide are women [1]. In spite of the differences between the two countries, results showed that women in both places are highly vulnerable to the effects of unexpected prolonged stress situations. Aggravation of chronic pain symptoms such as TMD and symptoms associated with bruxism may be only some of the negative consequences that affect women more severely than men [41,42].

Additional factors that were consistently identified as significantly predicting the TMD, AB, and SB (and/or the symptom aggravation) in the present studies were anxiety, worries of being contaminated by the virus, and concerns about the pandemic’s effect on physical or mental health (to slightly different extents in the two countries). In some instances, two additional factors were identified in the regression analyses: worries that the pandemic will affect the relationship with relatives and friends (in Israel) and the use of social media (but not TV or internet) for checking news regarding the pandemic (in Poland).

In Israel, close family ties and long-term friendships are very common in the society [43]. Apparently, the social distancing period, which prevented face-to-face meetings, took its toll on Israeli society. The fact that the use of social media affected, in some cases, the Polish, but not the Israeli, participants, may be explained by the younger age of the former. Another explanation may be that the Israeli society is constantly exposed to security tension and alerts making it more resilient [44]. The Israeli public extensively check the news at all times, and the Coronavirus crisis is no different from many other emergencies experienced by these people.

In a recent study, Varshney et al. reported that during the initial stages of the Coronavirus pandemic in India, almost one-third of the respondents manifested a significant psychological impact [45]. The factors that predicted a higher psychological impact were young age, female gender, and the presence of a physical comorbidity. The authors of the study also showed that males faced a lesser psychological impact as compared to females [45]. Thus, in spite of the differences between countries and cultures, many of the basic factors affecting the public are similar.

Several limitations of the studies should be pointed out. No inclusion and/or exclusion criteria were specified and the study samples were not predetermined. The significant differences in demographic variables might have been a reason for some of the detected differences, especially in view of the fact that gender (but not age) came out as a predictive factor in most of the models calculated for TMD, bruxism, and symptom aggravation, in both countries. Moreover, the studies were performed during a specific point in time at the first phase of the pandemic and may be indicative of the immediate stress evoked by the sudden health risk and changes in life style. Additionally, possible confounders that could have influenced the results were not under control.

Further longitudinal studies are needed to evaluate the pandemic’s possible long-term mental and physical consequences. Multifactorial and multicultural research should be performed to identify the risk groups and counteract the aggravation of emotional and physical effects in the case of future global crises.

## 5. Conclusions

The coronavirus pandemic has caused significant adverse effects on the psychoemotional status of both Israeli and Polish populations, resulting in the intensification of their bruxism and TMD symptoms and thus leading to increased orofacial pain.

## Figures and Tables

**Table 1:** Gender of study populations.

Gender	Percent Israel	Count	Percent Poland	Count
Male	33.6%	235	41.6%	454
Female	66.4%	465	58.4%	638
Total	100%	700	100%	1092

**Table 2:** Age of study populations.

Age	Israel			Poland		
	Female N (%)	Male N (%)	Total	Female N (%)	Male N (%)	Total
18–35	142 (30.5)	61 (26.0)	203	443 (69.4)	385 (84.8)	828
36–55	185 (39.8)	98 (41.7)	283	171 (26.8)	63 (13.9)	234
>56	127 (27.3)	73 (31.1)	200	24 (3.8)	6 (1.3)	30
N/A	11 (2.4)	3 (1.3)	14	0	0	0
Total	465	235	700	638	454	1092

**Table 3:** Temporomandibular disorders (TMD) distribution.

		TMD Positive		TMD Negative		*p* *	OR (95% CI) ^#^
Age	Gender	Israel	Poland	Israel	Poland		
18–35	Male N (%)	7 ((1.6)	158 (35.4)	54 (12.1)	227 (50.9)	0.0000	5.37 (2.38, 12.11)
	Female N (%)	48 (8.2)	280 (47.8)	94 (16.1)	163 (27.9)	0.0000	3.36 (2.26, 5.00)
36–55	Male N (%)	13 (8.1)	20 (12.4)	85 (52.8)	43 (26.7)	0.005	3.04 (1.38, 6.69)
	Female N (%)	47 (13.2)	105 (29.5)	138 (38.8)	66 (18.5)	0.0000	4.67 (2.9, 7.34)
>56	Male N (%)	10 (12.7)	1 (1.3)	63 (79.7)	5 (6.3)	>0.05	1.26 (0.13, 11.93)
	Female N (%)	25 (16.6)	12 (7.9)	102 (67.6)	12 (7.9)	0.003	4.08 (1.64, 10.16)
N/A		2	0	12	0	-----	----
Total		152	576	548	516		

* Comparison of countries in regard to TMD positive/TMD negative in particular age and gender groups (Chi^2^). ^#^ OR comparing Poland versus Israel in regard to TMD positive in particular age and gender groups.

**Table 4:** Awake bruxism (AB) distribution.

		Probable AB (I)		Possible AB (II)		AB Negative (III)		*p* *	OR (95% CI) ^#^
Age	Gender	Israel	Poland	Israel	Poland	Israel	Poland		
18–35	Male N (%)	8 (1.8)	71 (15.9)	21 (4.7)	138 (30.9)	32 (7.2)	176 (39.5)	>0.05	1.31 (0.76, 2.25)
	Female N (%)	40 (6.8)	187 (32.0)	38 (6.5)	151 (25.8)	64 (10.9)	105 (17.9)	0.0000	2.64 (1.78, 3.93)
36–55	Male N (%)	19 (11.8)	17 (10.6)	15 (9.3)	19 (11.8)	64 (39.7)	27 (16.8)	0.015	2.51 (1.31, 4.81)
	Female N (%)	46 (12.9)	94 (26.4)	38 (10.7)	50 (14.0)	101 (28.4)	27 (7.6)	0.0000	6.41 (3.88, 10.60)
>56	Male N (%)	8 (10.1)	0	4 (5.1)	1 (1.3)	61 (72.2)	5 (6.3)	>0.05	1.02 (0.11, 9.50)
	Female N (%)	30 (19.9)	9 (6.0)	9 (6.0)	6 (4.0)	88 (58.3)	9 (6.0)	0.007	3.76 (1.52, 9.33)
N/A		2	0	0	0	12	0	----	----
Total		153	378	125	365	422	349		

* Comparison of countries in regard to Possible/Probable AB/AB negative in particular age and gender groups (Chi^2^). ^#^ OR comparing Poland versus Israel in regard to AB positive (Possible and Probable AB) in particular age and gender groups.

**Table 5:** Sleep bruxism (SB) distribution.

		Probable SB (I)		Possible SB (II)		SB Negative (III)		*p* *	OR (95% CI) ^#^
Age	Gender	Israel	Poland	Israel	Poland	Israel	Poland		
18–35	Male N (%)	8 (1.8)	61 (13.7)	9 (2.0)	74 (16.6)	44 (9.8)	250 (56.0)	0.008	1.40 (0.77, 2.54)
	Female N (%)	34 (5.8)	182 (31.1)	21 (3.6)	90 (15.4)	87 (14.9)	171 (29.2)	0.0000	2.52 (1.71, 3.71)
36–55	Male N (%)	22 (13.7)	16 (9.9)	9 (5.6)	7 (4.4)	67 (41.6)	40 (24.8)	>0.05	1.24 (0.64, 2.42)
	Female N (%)	50 (10.7)	84 (23.6)	23 (6.5)	21 (5.9)	112 (31.5)	66 (18.5)	0.0000	2.44 (1.59, 3.74)
>56	Male N (%)	6 (7.6)	0	4 (5.1)	1 (1.3)	63 (79.8)	5 (6.3)	>0.05	1.26 (0.13, 11.93)
	Female N (%)	29 (19.2)	8 (5.3)	4 (2.7)	6 (4.0)	94 (62.2)	10 (6.6)	0.0008	3.99 (1.62, 9.84)
N/A		3	0	0	0	11	0	----	----
Total		152	351	70	199	478	542		

* Comparison of countries in regard to Possible/Probable SB/SB negative in particular age and gender groups (Chi^2^). ^#^ OR comparing Poland versus Israel in regard to SB positive (Possible and Probable SB) in particular age and gender groups.

**Table 6:** Prediction of temporomandibular disorders (TMD) in Poland.

Effect	Predictor	Estimate	S.E.	Wald	df	OR (95% CI)	*p*
Gender	Female	0.384	0.065	34.516	1	2.16 (1.67, 2.78)	0.0000
Risk of contamination *	Yes	0.237	0.065	13.526	1	1.61 (1.25, 2.07)	0.0002
Anxiety	Yes	0.372	0.082	20.505	1	2.10 (1.53, 2.90)	0.0000
Mental health **	II	0.160	0.069	5.354	1	1.38 (1.05, 1.80)	0.0207

Link function: Logit. * Feeling at high risk of being contaminated (yes/no). ** Worries about the effect of the Coronavirus on mental health (not at all/a little worried (I) versus somewhat worried/worried/very worried (II)).

**Table 7:** Prediction of temporomandibular disorders (TMD) aggravation in Poland.

Effect	Predictor	Estimate	S.E.	Wald	df	OR (95% CI)	*p*
Gender	Female	0.321	0.072	19.715	1	1.90 (1.43, 2.52)	0.0000
Risk of contamination *	Yes	0.218	0.069	10.150	1	1.55 (1.18, 2.03)	0.0014
Social media **	II	0.249	0.069	12.929	1	1.65 (1.25, 2.16)	0.0003
Anxiety	Yes	0.389	0.08	23.579	1	2.18 (1.59, 2.98)	0.0000
Mental health ***	II	0.224	0.073	9.372	1	1.57 (1.18, 2.09)	0.0022

Link function: Logit. * Feeling at high risk of being contaminated (yes/no). ** How often connecting to social media to check for news regarding the pandemic (not checking at all/checking once a day (I) versus checking several times a day/checking all the time (II)). *** Worries about the effect of the Coronavirus on mental health (not at all/a little worried (I) versus somewhat worried/worried/very worried (II)).

**Table 8:** Prediction of temporomandibular disorders (TMD) aggravation in Israel.

Effect	Predictor	Estimate	S.E.	Wald	df	OR (95% CI)	*p*
Gender	Female	0.255	0.127	4.041	1	1.66 (1.01, 2.74)	0.0444
Relations *	II	0.375	0.112	11.155	1	2.12 (1.36, 3.29)	0.0008
Anxiety	Yes	0.351	0.123	8.184	1	2.02 (1.25, 3.26)	0.0042

Link function: Logit. * Worries regarding the effect of the Coronavirus pandemic on relations with relatives and friends (not at all/a little worried (I) versus somewhat worried/worried/very worried (II).

**Table 9:** Prediction of awake bruxism (AB) in Poland.

Effect	Predictor	Estimate	S.E.	Wald	df	OR (95% CI)	*p*
Gender	Female	0.472	0.069	46.245	1	2.57 (1.96, 3.37)	0.0000
Risk of contamination *	Yes	0.212	0.070	9.089	1	1.53 (1.16, 2.01)	0.0026
Mental health **	II	0.249	0.075	11.041	1	1.64 (1.23, 2.21)	0.0009
Anxiety	Yes	0.334	0.095	12.215	1	1.95 (1.34, 2.83)	0.0005

Link function: Logit. * Feeling at high risk of being contaminated (yes/no). ** Worries about the effect of the Coronavirus on mental health (not at all/a little worried (I) versus somewhat worried/worried/very worried (II).

**Table 10:** Prediction of awake bruxism (AB) aggravation in Poland.

Effect	Predictor	Estimate	S.E.	Wald	df	OR (95% CI)	*p*
Gender	Female	0.349	0.074	22.300	1	2.01 (1.50, 2.69)	0.0000
Risk of contamination *	Yes	0.208	0.071	8.615	1	1.51 (1.15, 2.00)	0.0033
Anxiety	Yes	0.461	0.081	32.200	1	2.51 (1.82, 3.46)	0.0000
Physical health **	II	0.217	0.075	8.371	1	1.54 (1.15, 2.07)	0.0038
Mental health ***	II	0.260	0.076	11.781	1	1.68 (1.25, 2.26)	0.0006
Social media ****	II	0.241	0.071	11.516	1	1.62 (1.23, 2.14)	0.0007

Link function: Logit. * Feeling at high risk of being contaminated (yes/no). ** Worries about the effect of the Coronavirus on one’s physical health (not at all/a little worried (I) versus somewhat worried/worried/very worried (II)). *** Worries about the effect of the Coronavirus on one’s mental health (not at all/a little worried (I) versus somewhat worried/worried/very worried (II)). **** How often connecting to social media to check for news regarding the pandemic (not checking at all/checking once a day (I) versus checking several times a day/checking all the time (II).

**Table 11:** Prediction of awake bruxism (AB) in Israel.

Effect	Predictor	Estimate	S.E.	Wald	df	OR (95% CI)	*p*
Gender	Female	0.175	0.088	3.946	1	1.42 (1.00, 2.00)	0.0470
Depression	Yes	0.202	0.101	4.000	1	1.50 (1.01, 2.23)	0.0455
Finances *	II	0.233	0.081	8.283	1	1.59 (1.16, 2.19)	0.0040
Anxiety	Yes	0.383	0.109	12.472	1	2.15 (1.41, 3.30)	0.0004

Link function: Logit. * Worries about finances (not at al/a little worried (I) versus somewhat worried/worried/very worried (II)).

**Table 12:** Prediction of awake bruxism (AB) aggravation in Israel.

Effect	Predictor	Estimate	S.E.	Wald	df	OR (95% CI)	*p*
Gender	Female	0.333	0.134	6.208	1	1.95 (1.15, 3.29)	0.0127
Relations *	II	0.250	0.123	4.156	1	1.65 (1.02, 2.67)	0.0417
Anxiety	Yes	0.445	0.131	11.522	1	2.44 (1.46, 4.08)	0.0007
Mental health **	II	0.292	0.134	4.737	1	1.79 (1.06, 3.04)	0.0295

Link function: Logit. * Worries regarding the effect of the Coronavirus pandemic on relations with relatives and friends (not at all/a little worried (I) versus somewhat worried/worried/very worried (II)). ** Worries about the effect of the Coronavirus on one’s mental health (not at all/a little worried versus somewhat worried/worried/very worried).

**Table 13:** Prediction of sleep bruxism (SB) in Poland.

Effect	Predictor	Estimate	S.E.	Wald	df	OR (95% CI)	*p*
Gender	Female	0.485	0.065	55.413	1	2.64 (2.04, 3.41)	0.0000
Risk of contamination *	Yes	0.198	0.064	9.646	1	1.49 (1.16, 1.91)	0.0019
Anxiety	Yes	0.225	0.074	9.341	1	1.57 (1.18, 2.09)	0.0022

Link function: Logit. * Feeling at high risk of being contaminated (yes/no).

**Table 14:** Prediction of sleep bruxism (SB) aggravation in Poland.

Effect	Predictor	Estimate	S.E.	Wald	df	OR (95% CI)	*p*
Gender	Female	0.329	0.077	18.030	1	1.93 (1.42, 2.61)	0.0000
Risk of contamination *	Yes	0.301	0.072	17.302	1	1.83 (1.38, 2.43)	0.0000
Anxiety	Yes	0.405	0.083	24.071	1	2.25 (1.63, 3.11)	0.0000
Social media **	II	0.230	0.073	10.026	1	1.58 (1.19, 2.11)	0.0015
Mental health ***	II	0.245	0.078	9.939	1	1.63 (1.20, 2.21)	0.0016

Link function: Logit. * Feeling at high risk of being contaminated (yes/no)**.** ** How often connecting to social media to check for news regarding the pandemic (not checking at all/checking once a day (I) versus checking several times a day/checking all the time (II))**.** *** Worries about the effect of the Coronavirus on one’s mental health (not at all/a little worried (I) versus somewhat worried/worried/very worried (II).

**Table 15:** Prediction of sleep bruxism (SB) in Israel.

Effect	Predictor	Estimate	S.E.	Wald	df	OR (95% CI)	*p*
Anxiety	Yes	0.323	0.103	9.762	1	1.91 (1.27, 2.86)	0.0018
Relations *	II	0.359	0.091	15.516	1	2.05 (1.43, 2.92)	0.0001

Link function: Logit. * Worries regarding the effect of the Coronavirus pandemic on relations with relatives and friends (not at all/a little worried (I) versus somewhat worried/worried/very worried (II)).

**Table 16:** Prediction of sleep bruxism (SB) aggravation in Israel.

Effect	Predictor	Estimate	S.E.	Wald	df	OR (95% CI)	*p*
Gender	Female	0.419	0.147	8.160	1	2.31 (1.30, 4.11)	0.0043
Anxiety	Yes	0.358	0.139	6.665	1	2.05 (1.19, 3.53)	0.0098
Mental health *	II	0.346	0.131	6.971	1	2.00 (1.20, 3.34)	0.0083

Link function: Logit. * Worries about the effect of the Coronavirus on one’s mental health (not at all/a little worried (I) versus somewhat worried/worried/very worried (II))
